# The role of glucose metabolism in wound healing: an overview

**DOI:** 10.1093/burnst/tkaf053

**Published:** 2025-07-31

**Authors:** Tao Zhang, Youjing Yang, Junyu Jiang, Wenyu Du, Guangbin Huang, Dingyuan Du, Shasha Tao

**Affiliations:** Department of Trauma Surgery, Chongqing Emergency Medical Center, Chongqing University Central Hospital, School of Medicine, Chongqing University, No. 1, Jiangkang Road, Yuzhong District, 400014, Chongqing, China; Chongqing Key Laboratory of Emergency Medicine, Chongqing Emergency Medical Center, Chongqing University Central Hospital, No. 1, Jiangkang Road, Yuzhong District, 400014, Chongqing, China; Department of Trauma Surgery, Chongqing Emergency Medical Center, Chongqing University Central Hospital, School of Medicine, Chongqing University, No. 1, Jiangkang Road, Yuzhong District, 400014, Chongqing, China; Department of Trauma Surgery, Chongqing Emergency Medical Center, Chongqing University Central Hospital, School of Medicine, Chongqing University, No. 1, Jiangkang Road, Yuzhong District, 400014, Chongqing, China; Department of Trauma Surgery, Chongqing Emergency Medical Center, Chongqing University Central Hospital, School of Medicine, Chongqing University, No. 1, Jiangkang Road, Yuzhong District, 400014, Chongqing, China; Department of Trauma Surgery, Chongqing Emergency Medical Center, Chongqing University Central Hospital, School of Medicine, Chongqing University, No. 1, Jiangkang Road, Yuzhong District, 400014, Chongqing, China; Department of Trauma Surgery, Chongqing Emergency Medical Center, Chongqing University Central Hospital, School of Medicine, Chongqing University, No. 1, Jiangkang Road, Yuzhong District, 400014, Chongqing, China; Chongqing Key Laboratory of Emergency Medicine, Chongqing Emergency Medical Center, Chongqing University Central Hospital, No. 1, Jiangkang Road, Yuzhong District, 400014, Chongqing, China

**Keywords:** Glucose metabolism, Wound healing, Chronic wounds, Therapeutic potential

## Abstract

Glucose metabolism is the core process by which cells obtain energy, providing adenosine triphosphate and metabolic intermediates through glycolysis and the tricarboxylic acid cycle and supporting cell proliferation, migration, and functional maintenance. It not only fuels cells but also cranks out nicotinamide adenine dinucleotide phosphate (NADPH) via the pentose phosphate pathway. This NADPH is crucial for fending off oxidative stress, keeping immune responses in check, and playing a role in cell signaling. During the process of wound healing, glucose metabolism plays a crucial role in each stage. In the early stage, cells rely on glycolysis to generate energy for proliferation and migration; during the inflammatory phase, immune cells generate reactive oxygen species through glucose metabolism to eliminate pathogens; and during the proliferation and remodeling phase, glucose metabolism supports the generation of the extracellular matrix and tissue repair. However, in chronic wounds, abnormal glucose metabolism increases oxidative stress and inflammatory responses, significantly delaying wound healing. Understanding how abnormal glucose metabolism affects the wound microenvironment and cell function can help researchers develop new therapeutic strategies. Therefore, this review breaks down how glucose metabolism works at each stage of wound healing. We are highlighting its potential as something we can target therapeutically, and hoping to spark some fresh ideas and avenues for research and clinical use down the road.

Highlights

- Wound healing necessitates dynamic glucose metabolism shifts: glycolysis fuels early migration; oxidative phosphorylation drives later matrix synthesis. Chronic wounds exhibit metabolic dysfunction, suggesting “spatiotemporal regulation” as a therapy.
- Macrophage polarization (M1/M2) and glucose metabolism are interconnected. AMPK/STAT6 dysregulation in diabetic wounds impairs immune homeostasis, addressable via metabolic reprogramming.
- Enzymes (HK2/PFKFB3) and metabolites (lactate) exhibit dual therapeutic roles, promoting repair but potentially causing scar hyperplasia/inhibiting MMPs. Stage-specific interventions (e.g. PFKFB3 inhibition) and nanodelivery systems hold promise.
- Diabetic wounds display mitochondrial dysfunction (imbalanced dynamics, ROS), hindering healing. Mitochondrial transplantation or antioxidants (SkQ1) can restore metabolism and promote repair.
- Multi-pathway synergistic interventions, including traditional medicine and responsive materials, offer a comprehensive metabolic-immune-microenvironment approach for diabetic foot ulcers.

## Background

Wound care has long posed a significant challenge in the medical field. In the USA alone, chronic wounds affected >8 million individuals in 2014, with treatment costs exceeding $30 billion [1]. In the face of considerable progress in contemporary wound care, persistent wounds, particularly diabetic foot sores, bedsores, and those with infections, frequently take longer than 3 months to mend, thereby heightening the likelihood of dire consequences like sepsis, limb loss, or even fatality [2].

Wound healing constitutes a multifaceted physiological event reliant on orchestrated cellular and molecular interplay. Recent research highlights glucose metabolism as a key regulator of wound healing, influencing cell proliferation, migration, and immune responses beyond its fundamental role in the energy supply. These properties are especially relevant in diabetic patients, where abnormal glucose metabolism frequently leads to significantly delayed wound healing, making the recovery process more challenging. Therefore, a deeper investigation into the relationship between glucose metabolism and wound healing is crucial for optimizing current treatments and developing novel therapeutic strategies.

In mammalian cells, glucose serves as the primary energy source, and its metabolism is essential for maintaining cell growth, survival, and various functional activities. Aberrations in glucose metabolism are not only closely associated with impaired wound healing but also linked to the pathogenesis of numerous diseases. Distinct metabolic phenotypes characterize different wound healing stages, with early phases primarily relying on glycolysis to fuel rapid cell proliferation and migration [3, 4]. Additionally, glucose metabolism influences immune responses by regulating the production of reactive oxygen species (ROS), which play dual roles in wound healing—eliminating pathogens under normal conditions but exacerbating chronic inflammation when dysregulated, particularly in diabetic patients [5–7]. In this scenario, the persistent inflammatory state further complicates tissue repair and increases the risk of wound-related complications. This complex pathophysiological process underscores the dual role of glucose metabolism in wound healing, which serves both as an essential energy source and, when dysregulated, as a potential barrier to effective healing.

This article provides a comprehensive review of recent mechanistic studies on how glucose metabolism influences wound healing, explores the impact of glucose metabolism on the healing process, and discusses the development of innovative therapeutic strategies based on glucose regulation. These advances provide new insights into the biological underpinnings of wound repair and hold significant potential for the future development of more effective treatments. By delving deeper into the roles of glucose metabolism and its regulatory mechanisms in wound healing, researchers have hope for the development of more targeted therapies, ultimately improving patient outcomes and quality of life.

## Review

### Wound-healing process

**Figure 1:**
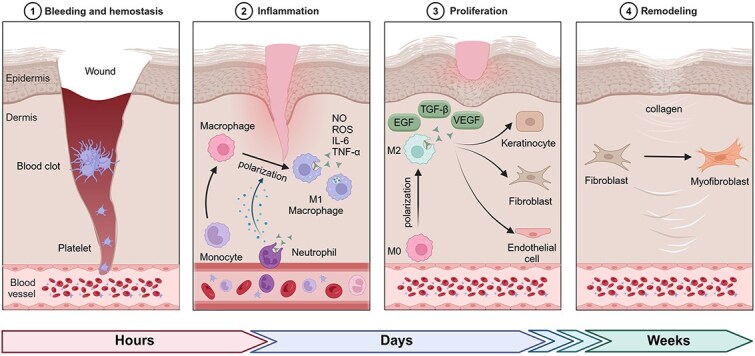
The four key phases of wound healing along with their approximate timing: (1) hemostasis (within minutes to hours post-injury), where clot formation halts bleeding through platelet activation; (2) inflammation (hours to days), characterized by neutrophil and macrophage infiltration, with M1 macrophages releasing proinflammatory mediators like NO, ROS, IL-6, and TNF-α; (3) proliferation (days to weeks), during which M2 macrophages promote tissue repair by secreting growth factors (TGF-β, EGF, VEGF) to stimulate keratinocytes, fibroblasts, and endothelial cells; and (4) remodeling (weeks to months), where fibroblasts differentiate into myofibroblasts and deposit collagen, forming scar tissue and restoring wound strength. This figure was created with BioRender (https://biorender.com)

Skin wound healing is a complex, dynamic, and highly organized biological process that encompasses the evolutionary development of genetic, epigenetic, and molecular mechanisms to achieve the spatial and temporal coordination of various cells [8]. This methodology is segmented into four key phases: hemostasis, the inflammatory response, cell growth, and tissue reconstruction [7, 9] (Figure 1). These processes rely on the rapid adjustment of energy metabolism and the precise regulation of cellular signaling.

When tissue gets damaged, the clotting process kicks into high gear, quickly forming a temporary mesh of fibrin that acts as a framework for cell movement. This matrix also guards the wound against harmful invaders [10]. During this phase, glucose metabolism supplies energy to platelets and endothelial cells, supporting their swift response. Through glycolysis, these cells generate the adenosine triphosphate (ATP) necessary to promote platelet aggregation and vasoconstriction, thereby effectively establishing an initial barrier.

The recruitment and activation of immune cells mark the onset of the inflammatory phase. Inflammatory cells such as neutrophils and macrophages rely on glucose metabolism to obtain energy, supporting their phagocytic activity and cytokine secretion. Both glycolysis and oxidative phosphorylation provide the necessary energy and metabolic intermediates for these cells, enabling them to eliminate pathogens and necrotic tissue while also releasing cytokines to regulate the subsequent healing process [11].

The proliferation and migration of fibroblasts and endothelial cells are key characteristics of the proliferative phase [12]. Glucose metabolism is critical in this stage, as cells require substantial energy to support rapid proliferation and matrix synthesis. Moreover, the energy supply of macrophages shifts from glycolysis to oxidative phosphorylation, inducing the M2 polarization of macrophages, which suppress inflammation and release growth factors to promote wound healing [13].

Finally, the ultimate goal of the tissue remodeling phase of wound healing is to restore the structure and function of the tissue. Glucose metabolism continues to play an essential role in this stage, supporting fibroblast-driven collagen synthesis and matrix reorganization. Type III collagen is progressively supplanted by type I collagen, enhancing tissue tensile resilience [14], with glucose metabolism providing the necessary energy and substrates for this process. Moreover, the apoptosis and removal of cells are also regulated by glucose metabolism, ensuring timely cellular clearance to maintain tissue homeostasis [15].

**Figure 2:**
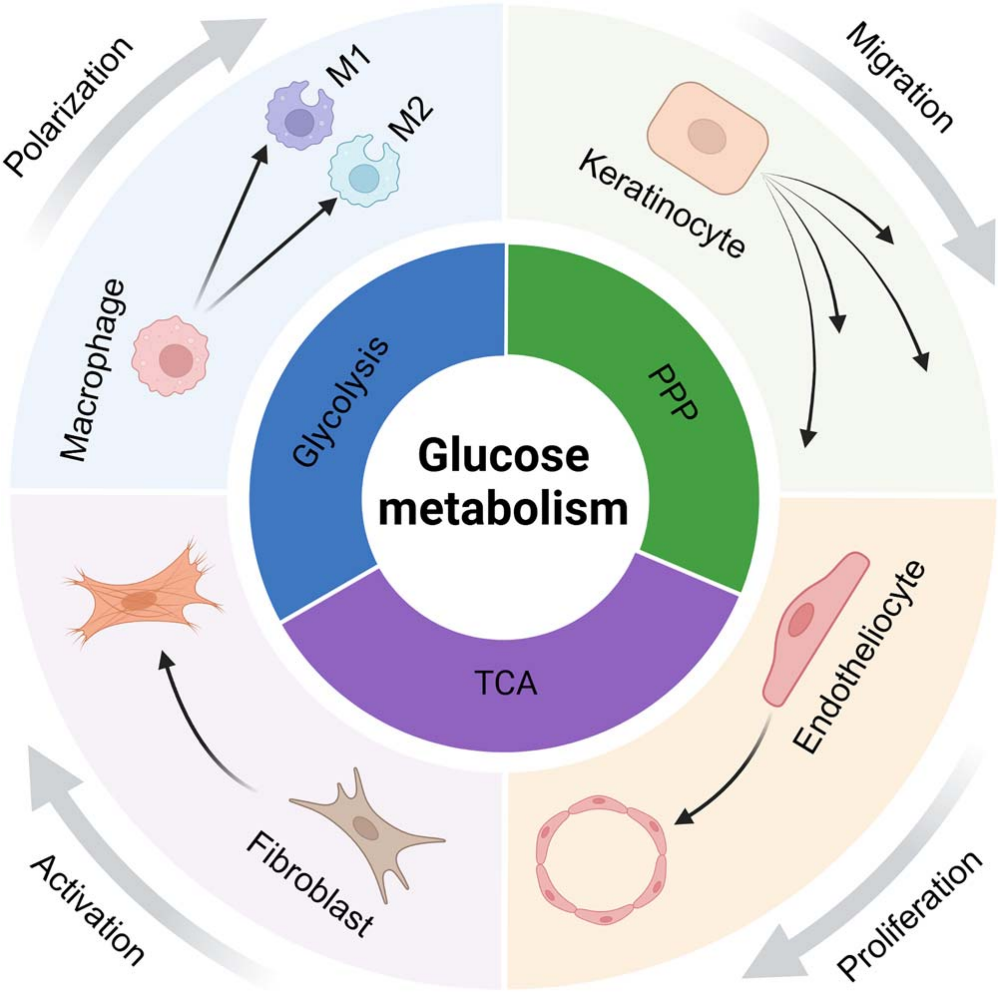
Glucose affects macrophage polarization, keratinocyte, fibroblast, and endothelial cell proliferation, migration, and activation through different metabolic pathways, such as glycolysis, the pentose phosphate pathway (PPP), and the tricarboxylic acid cycle (TCA), participating in the regulation of wound healing. This figure was created with BioRender (https://biorender.com)

Undoubtedly, wound healing involves intricate crosstalk between cells, factors, tissues, and vascular systems, with each phase providing signals that drive the next phase and glucose metabolism actively facilitating these transitions (Figure 2). However, research into the exact mechanisms involved remains insufficient, necessitating further clarification in future studies.

### Basic processes of glucose metabolism

Clarifying the role of glucose metabolism in wound healing first requires an understanding of the fundamental processes of glucose metabolism. Glucose is transported into cells via glucose transporters (GLUTs) and enters the glycolytic pathway. This process occurs in the cytoplasm, where key enzymes such as hexokinase (HK) and phosphofructokinase (PFK) breakdown glucose into pyruvate, producing ATP, which rapidly supplies energy for the various cellular activities essential for wound repair. Glycolysis is particularly critical in the early stages of acute wound healing, as it swiftly meets the energy demands of proliferating and migrating epithelial cells and fibroblasts [4]. With ample oxygen, pyruvate is then delivered to the mitochondria where it is transformed into acetyl-CoA via the action of the pyruvate dehydrogenase complex. Acetyl-CoA is then incorporated into the tricarboxylic acid (TCA) cycle, where subsequent enzymatic processes produce substantial levels of nicotinamide adenine dinucleotide (NADH) and flavin adenine dinucleotide (reduced form, FADH_2_). These electron carriers subsequently participate in oxidative phosphorylation via the electron transport chain, producing substantial amounts of ATP. This abundant energy supply is especially crucial during the proliferative and regenerative phases of wound healing [16]. Optimizing mitochondrial function is essential for maintaining efficient energy production and metabolic stability, which support tissue regeneration and wound repair. However, under hypoxic or low-oxygen conditions, particularly in chronic wounds such as diabetic foot ulcers, the limited oxygen availability restricts mitochondrial oxidative phosphorylation. Under these conditions, pyruvate is reduced to lactate by lactate dehydrogenase (LDH), generating ATP via anaerobic glycolysis. Although this process produces significantly less ATP than aerobic metabolism, it still provides a crucial energy source for cells in hypoxic environments, helping to sustain basic metabolic functions and support wound maintenance [17]. However, lactate accumulation may lead to local acidification, further impairing the wound-healing process.

**Figure 3:**
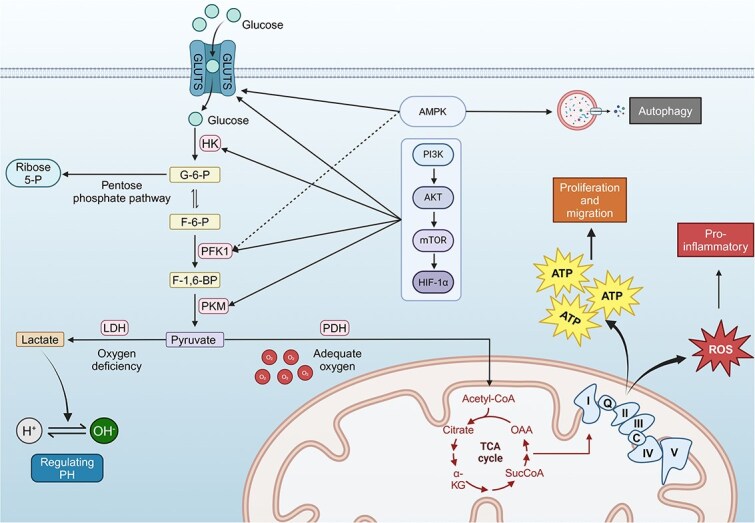
The molecular mechanism of glucose metabolism in wound healing. During wound healing, cells increase glucose uptake via GLUT transporters, activating glycolysis through key enzymes such as hexokinase (HK), phosphofructokinase-1 (PFK1), and pyruvate kinase (PKM). This generates ATP to fuel cell proliferation and migration, which are crucial for tissue repair. Lactate, produced by lactate dehydrogenase (LDH) under hypoxic conditions, helps regulate the wound pH, creating an acidic environment that facilitates healing. AMPK activation plays a vital role in enhancing glucose metabolism by promoting glycolytic flux and autophagy, ensuring cells meet their energy demands. Additionally, reactive oxygen species (ROS) produced during this process contribute to the proinflammatory response needed in the inflammatory phase. The PI3K–AKT–mTOR-HIF-1α pathway further supports glycolysis, promoting tissue regeneration and angiogenesis. The pentose phosphate pathway (PPP) provides essential biosynthetic precursors, aiding in cellular repair. This figure was created with BioRender (https://biorender.com)

Glycolysis and the TCA cycle, along with pathways like the pentose phosphate pathway (PPP), are crucial for glucose metabolism and wound repair. The PPP generates NADPH, which provides reducing power for antioxidant reactions, thereby mitigating oxidative stress and protecting cells from oxidative damage. It also produces nucleotide precursors that are crucial for DNA repair and cell proliferation [17]. In the wound microenvironment, oxidative stress is often elevated, and NADPH production is particularly vital for counteracting ROS-induced cellular damage. This process helps maintain cell viability and function, thereby promoting the healing process (Figure 3).

### Mechanisms of glucose metabolism in wound healing

#### The roles of key enzymes in glucose metabolism

During the wound-healing process, glycolysis and glucose uptake are upregulated, with multiple key enzymes involved in glucose metabolism undergoing changes to regulate the functions of various cells [18, 19]. Hexokinase, the first enzyme in glycolysis, not only promotes glucose phosphorylation but also modulates mitochondrial function, which are critical for keratinocyte migration and proliferation during wound healing [20]. Phosphofructokinase, a rate-limiting enzyme, influences the flux of glycolytic intermediates that support angiogenesis [21]. Pyruvate kinase, the final enzyme in glycolysis, regulates pyruvate production, which is essential for angiogenesis and tissue repair [22]. Chronic wounds are often accompanied by metabolic reprogramming [23, 24]. Therefore, analyzing the roles of glucose metabolism-related enzymes and their detailed molecular mechanisms during wound healing may help identify new therapeutic approaches to promote wound repair.

**Glucose transporters (GLUTs)** serve as essential carriers for the transport of glucose into cells and are widely distributed across various tissues. Glucose is taken up by GLUTs located on the cell membrane, ensuring the maintenance of glucose metabolism balance within the body. Numerous studies have shown a significant role for the GLUT family in the wound-healing process.

After injury, GLUT1 mRNA and protein expression rapidly increase, which enhances the efficiency of glucose transport and provides the metabolic energy necessary for cell migration and proliferation [25]. In keratinocytes, glucose transport is primarily mediated by GLUT1. *In vitro*, keratinocytes lacking GLUT1 exhibit impaired metabolism, increased oxidative stress, and a significantly reduced proliferative capacity. Moreover, the specific deletion of GLUT1 in mouse skin weakens the proliferation and migration of keratinocytes, thereby inhibiting wound healing [26]. However, the biological functions of GLUTs may vary across different cell types. For instance, a study conducted by Freemerman and colleagues demonstrated that heightened GLUT1 expression in macrophages boosted cellular glucose absorption and metabolic activity, elevated concentrations of pentose phosphate pathway byproducts, and diminished oxygen consumption rates in mice on a high-fat diet. Additionally, macrophages overexpressing GLUT1 exhibit increased secretion of inflammatory mediators, indicating a heightened inflammatory state, which suggests that this proinflammatory response may be driven by glucose-mediated oxidative stress [27]. Yu *et al*. reported that GLUT1 expression is increased in macrophages following the induction of M1 polarization, whereas GLUT3 expression is upregulated after the induction of M2 polarization. During the healing of diabetic foot ulcers, nonhealing samples presented significantly lower GLUT3 expression than healing samples. In a mouse wound healing model, conditional deletion of GLUT3 in macrophages led to impaired M2 polarization, resulting in delayed wound healing [28]. Moreover, placental mesenchymal stem cells (MSCs) enhance hypoxic wound repair via insulin secretion and increased GLUT1/2/3/adhesion molecule expression [29]. GLUT2 and GLUT4 are pivotal in modulating glucose transport throughout wound recovery, facilitating wound repair [30]. However, sustained activation of GLUTs potentially fosters dermatological conditions. In people affected by prevalent chronic inflammatory dermatoses like psoriasis or eczema, GLUT1 expression is increased in proliferating keratinocytes [4] and is significantly upregulated in hypertrophic scars [31].

GLUTs mediate both tissue repair (via cell growth and movement) and, potentially, detrimental inflammation contingent on specific factors. Therefore, the precise regulation of GLUT expression and activity requires a deeper understanding of the dependency and mechanisms by which different cell types respond to GLUTs. Additionally, a consideration of the variations in GLUT function in different physiological and pathological states is essential to ensure their optimal performance during the healing process. This information would enhance tissue repair and improve both patient recovery outcomes and quality of life.

**Hexokinase (HK)** is the rate-limiting enzyme in glycolysis, converting glucose to glucose-6-phosphate (G-6-P), a key metabolic intermediate involved in glycolysis, glycogen synthesis, and other pathways. These processes support cellular energy metabolism, glucose storage, and protein glycosylation, ensuring cellular adaptability and balance [32].

Hexokinase 2 (HK2), the most active isoenzyme, is found mainly in insulin-sensitive tissues. It promotes glycolysis by binding to VDAC1 on the mitochondrial membrane, increasing ATP production and preventing apoptosis [33]. HK2 is regulated by pathways such as the PI3K/Akt/HIF-1α, β-catenin/c-Myc, STAT3, and miR-199a pathways [34–37].

Studies have shown that HK activity is significantly reduced in the wound tissues of aging rats and diabetic rats [38, 39]. Inhibiting HK2 expression in keratinocytes significantly reduces cell proliferation and the glycolytic capacity [20, 40], suggesting that HK2 may play a protective role in wound healing. However, a study by Li *et al*. revealed that inhibiting hexokinase activity in macrophages reduces pyruvate accumulation and redirects it toward acetyl-CoA formation, thus suppressing glycolysis and normalizing the TCA cycle to alleviate inflammation, which in turn promotes wound healing [41]. In fibroblasts, high expression of HK promotes cell proliferation and migration, leading to scar hyperplasia [42].

In summary, HK2 regulates glucose metabolism and cell proliferation, particularly during wound healing. However, its effects vary across different cell types, warranting further research to better modulate HK2 for targeted therapies and improve wound healing outcomes.

**Phosphofructokinase-1 (PFK1)** is a key regulatory enzyme in the glycolytic pathway, and its activity is crucial for regulating the equilibrium of cellular energy processes. PFK1 catalyzes the reaction between fructose-6-phosphate (F-6-P) and ATP to produce fructose-1,6-bisphosphate (F-1,6-BP) [43]. PFK1 activity is regulated by the G6P concentration. Therefore, G-6-P produced by HK can indirectly modulate the activity of PFK1, thereby influencing the glycolysis rate and playing a synergistic role in wound healing. In its monomeric form, PFK1 is unstable, but it can maintain partial catalytic activity upon forming dimers [44]. PFK1’s tetrameric configuration ensures full activity, significantly regulating the pace of glycolysis [45].

Research has shown that increased PFK1 activity can increase glycolytic rates and cell proliferation, which may have positive effects on wound healing [46]. Interestingly, in diabetic wounds, PFK1 is often hyperactivated [39]; however, this increased activity does not necessarily lead to improved wound healing. This seemingly paradoxical phenomenon suggests that the role of PFK1 in wound healing may be more complex and could be regulated by other factors unique to the pathological conditions of diabetes. Future studies should address several key questions: first, the specific mechanisms underlying the elevated PFK1 activity in diabetic wounds need to be clarified; second, how changes in PFK1 activity interact with other metabolic pathways and cellular signaling networks to affect wound healing should be explored; and third, the feasibility of developing new therapeutic strategies by modulating PFK1 activity to enhance diabetic wound recovery should be evaluated. An in-depth investigation of these issues holds significant theoretical and practical value to comprehend the underlying processes of tissue repair and innovate new treatment strategies.

**Pyruvate kinase (PK)** is the terminal enzyme in the glycolytic sequence, facilitating the transformation of phosphoenolpyruvate (PEP) and adenosine diphosphate (ADP) into pyruvate and ATP. PK consists of several isoenzymes, with muscle-type pyruvate kinase (PKM) further divided into two subtypes, PKM1 and PKM2 [47]. PKM2 is a highly versatile enzyme whose activity depends on its oligomeric state. In its tetrameric form, PKM2 exhibits high catalytic activity, efficiently converting PEP to pyruvate and significantly increasing glycolytic flux, thus providing the ATP necessary for rapid cell proliferation [48]. This high-activity conformation is particularly important in rapidly proliferating cells. In contrast, when PKM2 exists in its monomeric or dimeric form, it can enter the nucleus and function as a coactivator for several transcription factors (such as HIF-1α, β-catenin/c-Myc, NF-κB, and STAT3), regulating metabolic reprogramming and gene expression to meet various physiological demands [49].

Emerging research highlights the pivotal role of PKM2 in wound recovery processes. During the early inflammatory phase of wound healing, PKM2 expression is significantly upregulated, particularly in proliferating keratinocytes. This upregulation not only enhances glycolytic flux, providing abundant ATP and metabolic intermediates for the cells, but also enhances angiogenesis through upregulation of vascular endothelial growth factor (VEGF) expression, supplying oxygen and nutrients to the regenerating tissue, and accelerating wound healing [22]. Moreover, PKM2 is pivotal in modulating inflammatory reactions; it triggers the AMPK signaling route, fostering the differentiation of macrophages into the M2 anti-inflammatory type [50]. M2 macrophages exhibit tissue-reparative and anti-inflammatory functions via cytokine/growth factor secretion, promoting regeneration and limiting inflammation. By modulating macrophage polarization, PKM2 helps maintain balance in the inflammatory response during wound healing, preventing excessive inflammation and thus promoting recovery. Moreover, PKM2 plays a key role in controlling cell growth and viability. For example, PKM2 promotes the proliferation and survival of intestinal epithelial cells by activating the Wnt/β-catenin signaling pathway while suppressing excessive inflammation [51]. This mechanism may similarly apply to skin wound repair, aiding in the restoration of the function of the damaged tissue.

In conclusion, PKM2 plays a critical role in wound healing through multiple regulatory mechanisms. It not only contributes to energy metabolism and the supply of biosynthetic materials but also regulates inflammatory responses and cell proliferation through signaling pathways. Further investigations into the specific mechanisms of PKM2 in wound healing will provide valuable insights for developing new therapeutic strategies, speeding up the healing and renewal of injured tissues.

**Lactate dehydrogenase (LDH)** is typically composed of two subunits, LDHA and LDHB, which form homotetrameric or heterotetrameric complexes in various combinations [52]. In the glycolytic pathway, LDH plays a critical role by driving pyruvate’s conversion to lactate coupled with NADH’s oxidation into NAD+, thus maintaining glycolytic flux and supporting cell survival under hypoxic conditions [53]. PK and LDH interact in the final stages of glycolysis and anaerobic metabolism. PK converts PEP to pyruvate, whereas LDH converts pyruvate to lactate under anaerobic conditions. This interplay ensures ATP production in low-oxygen environments, such as during wound healing. However, the two subunits of LDH have distinctly different functions. Under aerobic conditions, LDHB primarily mediates pyruvate regeneration from lactate, allowing pyruvate to enter the TCA cycle, thereby increasing mitochondrial ATP production, and promoting efficient energy utilization [54]. In contrast, under hypoxic conditions, LDHA has a greater affinity for pyruvate, converting it to lactate while regenerating NAD^+^ from NADH and supporting anaerobic glycolysis [52, 54].

Historically, lactate has been classified as a metabolic byproduct. Yet, contemporary research has shifted perspectives on lactate’s functions in health and disease states. As the end product of glycolysis, lactate serves a multitude of crucial functions in biology, such as managing energy metabolism, shaping memory, aiding in the mending of wounds, aiding tissue recovery from ischemia, and influencing the growth and movement of cancer cells [55]. In chronic wounds, the hyperactivity of matrix metalloproteinases (MMPs) is considered a key factor that delays healing. Research indicates that applying acidic wound dressings to shift the wound environment from alkaline to acidic can significantly suppress MMP activity. This adjustment in pH levels not only helps move the healing process from inflammation to proliferation but also promotes faster blood vessel formation [56].

Recent studies have highlighted lactate’s surprising importance in tissue repair. When lactate builds up in damaged areas, it triggers a biochemical shift—reducing carbon dioxide while boosting oxygen levels—which slightly acidifies the microenvironment. This carefully balanced acidity, falling within biologically safe parameters, actually stimulates cellular growth and specialization during the critical initial phase of wound recovery [57]. Additionally, wound formation activates the immune system, generating high energy demands, and lactate serves as a crucial energy substrate to meet the elevated metabolic needs for cell proliferation and repair during the healing process [58]. Research has also shown that an exogenous lactate intervention, such as the release of lactate from poly (lactic-co-glycolic acid) polymers, can significantly accelerate angiogenesis and wound repair, further supporting the positive role of lactate in wound healing [59, 60].

While the role of LDH in wound healing has not been extensively studied, its known functions in regulating lactate metabolism and the cellular redox state suggest potential contributions to tissue repair. For example, LDH activity may influence fibroblast proliferation, macrophage polarization, and endothelial cell migration by modulating lactate levels and the NAD^+^/NADH ratio. Further research should clarify LDH’s precise role in tissue repair and assess its viability as a treatment strategy.

As research into the roles of lactate in cell signaling and posttranslational modifications has intensified, its potential as a therapeutic strategy has gradually been revealed. For example, the involvement of lactate in regulating intracellular signaling pathways, modulating immune responses, and promoting tissue regeneration provides new insights and directions for chronic wound healing. Further research should focus on exploring the dual role of lactate as a signaling molecule and metabolic regulator, aiming to develop innovative therapeutic strategies targeting lactate metabolic pathways to provide more effective clinical interventions for chronic wounds and other related pathological conditions.

#### Pathways related to glucose metabolism

During the wound-healing process, various cells regulate glucose metabolic pathways to modulate inflammation and cell proliferation in a complex manner. This process involves not only the balance of energy supply and demand through metabolism but also the fine-tuning of cell signaling and gene expression. Glucose metabolism regulation is intricately tied to a host of vital signaling pathways. These pathways are pivotal in sustaining tissue homeostasis and in driving cell movement, growth, and development. Additionally, the metabolic products of glucose can act as signaling molecules, directly participating in the regulation of cellular immune responses and inflammation levels. Given the complexity and importance of these mechanisms, the following sections delve into these key signaling pathways and their roles in wound healing (Figure 3).

##### The tricarboxylic acid cycle

The TCA cycle is a central process in aerobic metabolism that oxidizes nutrients to generate ATP for cellular use. The TCA cycle not only provides energy but also contributes to the production of metabolic intermediates required for cellular biosynthesis. Its role in wound healing is particularly important in terms of the energy supply, synthesis, and redox regulation in cells.

The TCA cycle generates ATP through the oxidation of pyruvate, supporting the cellular energy demands. During wound healing, proliferating cells such as fibroblasts and keratinocytes require substantial energy for proliferation, migration, and metabolic activity. The TCA cycle ensures that cells have a steady energy supply, particularly during the proliferation and repair phases of wound healing [16]. TCA cycle intermediates such as citrate and oxaloacetate are not only crucial for energy metabolism but also play roles in the synthesis of fatty acids, amino acids, and other molecules required for cell growth and repair. During wound healing, these intermediates provide building blocks for new tissue formation, especially during the regeneration of skin and other tissues at the wound site [61, 62]. Oxidative stress is a significant factor in wound healing, particularly during the inflammatory phase. The regulation of the TCA cycle helps cells cope with excessive oxidative damage, maintaining cellular function and preventing metabolic dysfunction. By modulating the TCA cycle, cells can maintain the redox balance, thus promoting tissue repair and regeneration [63].

The TCA cycle is pivotal in wound recovery, furnishing essential energy and biosynthetic precursors for cellular growth and restoration. Furthermore, its regulation helps maintain the redox balance in cells, allowing them to better manage oxidative stress during the healing process. The TCA cycle ensures that cells can generate energy and synthesize essential molecules for wound repair, thereby supporting the overall healing process.

##### The pentose phosphate pathway

The PPP serves as a pivotal component of glucose metabolism and is indispensable for sustaining cellular redox equilibrium and facilitating tissue repair. This pathway generates NADPH and pentose sugars, which are vital for cell proliferation, DNA synthesis, and antioxidant defenses, especially during the various stages of wound healing.

The NADPH produced by the PPP provides a robust antioxidant defense for cells, which is particularly crucial at the wound site where free radicals are produced by immune cells and other factors. NADPH is a substrate for glutathione reductase, which helps convert harmful substances such as hydrogen peroxide into water, thus reducing oxidative damage and protecting cells and tissues from injury [64–66]. Pentose sugars, such as ribose-5-phosphate, are essential for nucleic acid synthesis. During wound healing, proliferating cells such as fibroblasts and keratinocytes require large amounts of nucleotides to synthesize DNA and RNA, ensuring that cell proliferation and tissue regeneration proceed efficiently [67]. The PPP is tightly linked to glycolysis and the TCA cycle. While the PPP provides NADPH and pentose sugars, it also influences cellular energy metabolism, helping cells adapt more efficiently to hypoxic and stress conditions. During wound healing, cells not only need energy but also require metabolic balance and functional diversity to respond effectively to injury.

The PPP supports wound healing by providing NADPH and pentose sugars, which are crucial for the antioxidant defense and cell proliferation. The interaction of the PPP with glycolysis and the TCA cycle further coordinates cellular energy metabolism and biosynthesis, offering essential metabolic support for the wound-healing process.

##### PI3K– AKT–mTOR signaling

AKT, a key serine/threonine kinase, regulates glucose metabolism, cell proliferation, and survival through PI3K activation [68]. The downstream protein mTOR functions in two complexes (mTORC1 and mTORC2) to modulate glycolysis by controlling GLUT expression and glycolytic enzymes [69, 70].

In a study of wound healing, Gao *et al*. [71] observed diverse changes in the levels of PI3K, AKT, phosphorylated PI3K (p-PI3K), and phosphorylated AKT (p-AKT) in injured mouse skin. The expression levels of p-PI3K and p-AKT peaked during the inflammatory and proliferative phases, whereas the expression of PI3K and AKT peaked during the remodeling phase. Hoke and colleagues [72] demonstrated a marked upregulation in both mRNA expression and protein concentrations of key components within the PI3K/AKT/mTOR signaling cascade during different phases of cutaneous wound repair in murine models. These findings underscore the pivotal involvement of this molecular pathway in orchestrating tissue regeneration. Additionally, Xiao *et al*. [73] reported that ozone oil could enhance the migration of fibroblasts and the epithelial–mesenchymal transition during wound healing by activating the PI3K/AKT/mTOR pathway. Huang *et al*. [74] discovered that in both nondiabetic and diabetic rat wound tissues, the AKT/mTOR pathway was activated, with significant increases in the expression of total proteins and key phosphorylated proteins. However, compared with those in nondiabetic wounds, the expression levels of these proteins were lower in diabetic wounds, suggesting that under diabetic conditions, the function of the PI3K/AKT pathway is impaired, leading to reduced uptake of 2-deoxyglucose and decreased expression of the GLUT1 transporter. These changes ultimately result in impaired glucose metabolism and delayed wound healing [75].

These findings collectively highlight the central role and multifaceted functions of the PI3K/AKT/mTOR signaling pathway in regulating the wound-healing process. Building on previous research, further exploration into how to optimize the activation or inhibition of this pathway to promote efficient wound healing is warranted. For example, addressing the functional impairments of the PI3K/AKT pathway to restore normal glucose metabolism in diabetic patients may be a key area for future investigations. Additionally, since AKT and its downstream mTOR complexes regulate several nodes involved in cell proliferation and angiogenesis, precise modulation of these nodes could facilitate a more refined approach to wound healing management.

Moreover, although existing studies indicate that the activation of the PI3K/AKT/mTOR pathway positively influences wound healing, the potential consequences of prolonged activation—such as excessive cell proliferation or other adverse effects like scar formation or an increased risk of tumorigenesis—remain to be further examined. Addressing these concerns will aid in optimizing therapeutic strategies based on the PI3K/AKT/mTOR pathway, ensuring their safety and efficacy in clinical applications.

##### Hypoxia-inducible factor-1 alpha

Hypoxia-inducible factor (HIF) is a cellular oxygen detector made up of an inducible HIF-α and a stable HIF-β subunit. Under hypoxic conditions, HIF maintains ATP homeostasis and regulates ROS production by modulating the TCA cycle and NADPH oxidase [76]. As a downstream effector of mTOR, HIF-1α plays key roles in metabolic reprogramming and phenotypic adaptation during wound healing [77, 78].

During the healing of wounds, the HIF-1α signaling pathway influences the metabolic state and functions of cells through various mechanisms. Upon sensing pathogen invasion, keratinocytes activate HIF-1α signaling, promoting IL-1β production and inducing aerobic glycolysis to meet the metabolic demands of the anti-infection response [79]. Even under normoxic conditions, activated HIF-1α can drive a shift in cellular metabolism toward aerobic glycolysis, driving the transition of immune cells into a proinflammatory state and further triggering mTOR pathway activation and HIF-1α upregulation [80]. This regulatory mechanism underscores the central role of HIF-1α in modulating cellular glycolysis, primarily by controlling glycolysis-associated gene expression, such as GLUT1, HK2, and PFKFB3, highlighting its critical role in metabolic control [81]. Research by Dhamija *et al*. [82] further showed that after HIF-1α activation, human primary keratinocytes exhibit increased glycolysis and significantly increased cell proliferation, indicating the important role of HIF-1α in regulating wound cell metabolism and proliferation.

HIF-1α significantly impacts the expansion, dissemination, and vascular development of endothelial cells in low-oxygen environments. In diabetic patients, hyperglycemic states can impair the adaptive cellular response to hypoxia, severely affecting the functionality of various cellular compartments during the wound-healing process [83]. Contemporary research suggests endothelial cells utilize glycolysis to fulfill energy needs during angiogenesis within ischemic tissues. The upregulation of HIF-1α increases the expression of GLUT1 and glycolytic enzyme-encoding genes. The use of chemical inhibitors to reduce HIF-1α expression significantly decreases glycolysis levels and the angiogenic capacity of endothelial cells *in vitro*, further confirming the critical role of HIF-1α in the regulation of wound healing [84].

Numerous investigations show HIF-1α considerably boosts the healing of injuries via fostering the growth of endothelial cells, keratinocytes, and fibroblasts [85–87]. However, the immune response and tissue repair mechanisms in skin injury require a delicate balance to ensure effective tissue regeneration while preventing infection. For example, the infiltration of natural killer (NK) cells under hypoxic conditions represents a HIF-mediated adaptive response to hypoxia. Research by Michal Sobecki *et al*. [88] showed that NK cells lacking HIF-1α exhibited impaired release of cytokines, such as interferon-gamma and granulocyte–macrophage colony-stimulating factor, in mouse models, potentially leading to delayed immune responses and accelerated skin angiogenesis and wound healing. However, this rapid healing may have been accompanied by a reduced bactericidal capacity and weakened systemic defenses against infection. In contrast, the activation of the HIF pathway supports the release of cytokines, enhancing NK cell–mediated antibacterial defenses, including direct cytotoxicity, but may delay wound closure. This study suggested that HIF-1α signaling in NK cells is a key node for coordinating skin antibacterial defenses and tissue repair.

In summary, HIF-1α supports wound healing in different cell types by regulating glycolytic pathways and promoting metabolic reprogramming to adapt to hypoxic environments, as well as modulating immune responses and cellular functional states. HIF-1α not only acts in keratinocytes, endothelial cells, and fibroblasts by enhancing glycolysis and angiogenesis but also coordinates antibacterial defenses and tissue regeneration in immune cells, collectively ensuring effective wound healing. Subsequent studies must delve deeper into the distinct processes of HIF-1α’s role in the healing of wounds across diverse cellular types, particularly in response to different oxygen conditions and pathological states. By obtaining deeper insights into the interactions between HIF-1α and other metabolic and signaling pathways, the overarching regulatory network involved in wound healing can be elucidated, aiding in the development of more precise intervention strategies that not only accelerate wound healing but also minimize unnecessary inflammatory responses and potential tissue damage.

##### Serine/threonine kinase AMP-activated protein kinase

AMP-activated protein kinase (AMPK) serves as a vital metabolic controller, pivotal in governing oxidative phosphorylation (OXPHOS). Its activation can promote a shift toward catabolic metabolism and reduce anabolic processes by phosphorylating various key proteins, thereby regulating lipid homeostasis, glycolysis, and mitochondrial stability [89]. Beyond directly controlling metabolic enzymes, AMPK also modulates the cell’s broader metabolic network through transcription factors [90]. The expansive regulatory duties of this system touch almost every aspect of an organism’s physiological and metabolic processes, especially by facilitating the movement of glucose transporters GLUT1 and GLUT4 to the cell membrane [91] and indirectly increasing glucose uptake by phosphorylating phospholipase D1 (PLD1) [92]. Furthermore, AMPK regulates the activity of the glycolytic rate-limiting enzyme PFK1 by phosphorylating PFKFB3, thereby impacting key steps in glucose metabolism [93].

In wounds of diabetic subjects, impaired AMPK activity correlates with protracted recovery, specifically impacting M2 macrophage differentiation and sustained glycolytic activation within these immune cells. Calycosin-7-glucoside (CG) enhances the mitochondrial membrane potential in M1 macrophages by activating the ROS/AMPK/STAT6 signaling cascade. This mechanism lowers the mitochondrial ADP/ATP ratio while curbing glycolysis, ultimately accelerating wound repair and stimulating the regeneration of granulation tissue. Essentially, CG fine-tunes macrophage metabolism to foster faster tissue recovery [94]. Additionally, activated AMPK can inhibit the mitochondrial reactive oxygen species (mtROS) production in diabetic wounds, significantly accelerating wound repair and improving angiogenesis. Increased oxidative stress in a high-glucose environment impairs the migration of skin fibroblasts and increases the generation of superoxide in the diabetic wound area [95]. Given the detrimental effects of oxidative stress on multiple cellular processes, activating AMPK to lower blood glucose levels and reduce ROS production could be vital for diabetic foot ulcer therapy [96–98].

Beyond its vital function in energy regulation, AMPK promotes tissue repair via autophagy modulation. Research indicates that autophagy-driven CCL2 production in keratinocytes is not just crucial for their movement and growth; it is also key to kicking dermal fibroblasts into gear. This further underscores autophagy’s pivotal role in the wound-healing process within living organisms [99]. AMPK modulates autophagy by phosphorylating multiple core components of autophagy pathways, featuring ULK1 (Ser467, Ser555, Thr574, Ser637), ATG9 (Ser761), VPS34 (Thr133, Ser135), Beclin1 (Ser91, Ser94), RACK1 (Thr50), and PAQR3 (Thr32) [100]. Moreover, the activation of the AMPK/ULK1 pathway has demonstrated the resumption of autophagy in keratinocytes, markedly enhancing the healing process of diabetic ulcers [101].

To sum up, AMPK acts as a versatile master switch in the wound-healing process, orchestrating metabolic balance and autophagy through complex pathways. Moving forward, investigations should delve deeper into how AMPK precisely modulates various cell types and disease conditions—especially in metabolic dysfunctions like diabetes, where its role could prove groundbreaking. By gaining deeper insights into how AMPK coordinates metabolic activities and cellular functions in various wound environments, we can provide a theoretical basis for developing new molecular-targeted therapeutic strategies to optimize clinical interventions for wound healing.

Additionally, the bridging role of AMPK in energy metabolism and autophagy regulation suggests its potential therapeutic value. Continued investigations into the downstream targets of AMPK and related signaling pathways will further clarify its specific roles in the wound-healing process. This study seeks to expedite the healing of wounds and mitigate complications stemming from metabolic disturbances, offering new directions and methods for clinical treatment.

##### Mitochondrial function

Mitochondria serve as cellular powerhouses, generating ATP through the mitochondrial respiratory chain and OXPHOS; ATP serves as the primary energy source for various physiological functions. Beyond their primary function in energy production, mitochondria are increasingly recognized for their vital roles in redox balance, cellular signaling, and programmed cell death [102]. In diabetic wounds, mitochondria exhibit significant structural and functional abnormalities, including reduced mitochondrial cristae, an altered morphology, and increased membrane permeability. These changes suggest a reduction in the functional units of mitochondria and a compromise in the integrity of the inner membrane [103]. Excess ROS levels are among the main triggers of mitochondrial dysfunction in diabetic wounds; they increase mitochondrial permeability, triggering cytochrome *c* liberation, thus initiating programmed cell death [104]. Reduced LDH activity diminishes the mitochondrial membrane potential, boosts ROS synthesis, and elevates Bax expression levels [52]. Additionally, in diabetic wounds, glucose metabolism is impaired due to insulin resistance and defective glucose uptake, which exacerbates the inhibition of mitochondrial oxidative phosphorylation and glycolysis, and leads to insufficient ATP production and subsequent mitochondrial dysfunction. This metabolic impairment, coupled with increased ROS production, disrupts wound healing by promoting chronic inflammation and oxidative stress [103].

Macrophages are essential for the healing process of wounds, as their metabolic state can rapidly adapt to changes in the microenvironment of damaged tissue, allowing them to switch their functional roles at different stages of healing. Both the early proinflammatory response and late pro-healing functions rely on the dynamic regulation of mitochondria. In diabetic wounds, an imbalance in mitochondrial homeostasis leads to excessive ROS production, compounded by defects in antioxidant defense mechanisms, which further result in cellular dysfunction and impede wound healing [83]. Revitalizing the mitochondrial membrane potential and functionality can reprogram macrophage metabolism, switching from glycolysis to oxidative phosphorylation in the mitochondria. This metabolic reprogramming promotes M2 macrophage polarization, which in turn helps mitigate persistent inflammation and accelerates tissue healing [105, 106].

In diabetic wounds, mitochondrial biogenesis is significantly inhibited. Transmission electron microscopy revealed a reduction in both the quality and quantity of mitochondria in the skin of diabetic mice, along with a marked decrease in mitochondrial transcription factor A (TFAM) levels [95]. Rizwan *et al*. further confirmed that in a high-glucose environment, TFAM mRNA expression in keratinocytes was reduced by nearly half [107]. This downregulation of TFAM provides evidence for weakened mitochondrial biogenesis, suggesting that the pharmacological activation of mitochondrial transcription to restore mitochondrial function may be an effective strategy to accelerate wound healing in patients with diabetes [108, 109].

Mitochondrial dynamics, specifically the equilibrium between fusion and fission, are crucial for maintaining mitochondrial functional integrity. Under diabetic conditions, increased mitochondrial fission and suppressed fusion lead to oxidative stress and functional impairments. Promoting mitochondrial fusion or inhibiting fission has been shown to correct this dynamic imbalance, reducing mtROS production and thereby improving mitochondrial function and wound healing [110–112]. Studies indicate that inhibiting the expression of regulatory proteins such as DRP1 can reverse mitochondrial fission, significantly reduce mtROS levels, and enhance mitochondrial health and angiogenesis [113, 114].

In summary, mitochondrial dysfunction in diabetic wounds results from a combination of factors, including energy metabolism disorders, excessive ROS production, limited mitochondrial biogenesis, and altered mitochondrial dynamics. While researchers have some understanding of these mechanisms, the complexity of their regulatory networks and interactions requires further investigation. Future research should focus on in-depth analyses of how mitochondrial function affects cellular behavior and wound healing across different wound types and healing stages. Additionally, regulating mitochondrial function, particularly through pharmacological and gene therapies, may provide new treatment strategies to improve healing in diabetic wounds. Interventions targeting mitochondrial function could accelerate wound healing and reduce complications associated with diabetes and other metabolic diseases. Continued research in this area will provide new theoretical foundations and practical directions for treating chronic wounds, with significant academic and clinical value.

### Therapeutic implications

The process of glucose metabolism is integral to the healing process of wounds, and zeroing in on glucose itself or pivotal proteins within those metabolic routes could potentially offer a powerful approach to speed up the healing process. A summary of some current therapeutic research on glucose metabolism in wound healing is provided below.

#### Regulating glucose in the diabetic wound microenvironment

The hyperglycemic microenvironment in diabetic wounds primarily arises from insulin resistance, insufficient insulin secretion, and disrupted glucose metabolism, which impair glucose uptake by cells and affect the energy supply [115]. Despite the elevated blood glucose levels, intracellular glucose utilization is restricted in diabetic wounds, resulting in suppressed glycolysis and mitochondrial respiration, as well as insufficient ATP production. Hyperglycemia also fosters the production of advanced glycation end products (AGEs), which activate chronic inflammation, further exacerbating oxidative stress and vascular dysfunction during the healing process [116]. Additionally, immune system dysfunction in diabetic wounds impairs the resolution of inflammatory responses, delaying wound healing. In contrast, glucose metabolism in nondiabetic wounds remains relatively normal, allowing cells to efficiently utilize glucose and generate ATP to support the healing process. Therefore, improving glucose metabolism in diabetic wounds, restoring the cellular energy supply, and alleviating chronic inflammation are critical for promoting wound healing.

Elevated glucose levels in blood and tissues are a significant characteristic of diabetic patients. Excess glucose not only enhances the virulence potential of *Staphylococcus aureus* but also weakens the antibacterial capacity because infiltrating phagocytes fail to express efficient glucose transporters, increasing the susceptibility of diabetic patients to bacterial infections [117]. These characteristics are primary contributors to compromised healing in diabetic patients’ wounds. Liang *et al*. [118] addressed this issue by designing a pH- and glucose-responsive metformin-releasing multifunctional hydrogel bandage. This bandage features enhanced adhesion, self-healing, easy removal, and antibacterial and antioxidant properties, as well as conductive hemostasis, aiding in the treatment of diabetic foot. Furthermore, a multifunctional, glucose-responsive metal–organic framework hydrogel was synthesized to catabolize surplus glucose into hydrogen peroxide and glucuronic acid for drug release [119]. This hydrogel not only alters the microenvironment of hyperglycemic wounds but also has synergistic antibacterial and angiogenic effects, facilitating diabetic wound repair.

In the high-glucose microenvironment of diabetic foot ulcers, excessive glycation reactions trigger chronic inflammation, further leading to delayed wound healing. Chen *et al*. [116] proposed a photothermal therapy approach that uses glucose in a high-glucose microenvironment as a substrate for sustainable hydrogen production and local glucose consumption to address this challenge; and this treatment inhibited the synthesis of advanced glycation end products and their receptor, thereby reducing skin cell apoptosis and promoting their proliferation and migration. Additionally, Shang *et al*. [120], Shi *et al*. [121], and Wang *et al*. [122] developed nanomaterials containing glucose oxidase, which can effectively lower local blood glucose levels, improve the microenvironment of chronic diabetic wounds, reduce bacterial infections, and promote wound healing. These studies suggest that by regulating the high-glucose environment and improving wound conditions, a significant enhancement of wound repair in diabetic patients can be achieved.

#### Regulation of signaling pathways

Patients with diabetes often face challenges in wound healing because of persistently high blood sugar levels. These hyperglycemic environments not only affect the local metabolic state of wounds but also trigger complex issues such as chronic inflammation, oxidative stress, and bacterial infections through various signaling pathways and cellular processes. Research shows that regulating glucose metabolism and targeting key signaling pathways can effectively improve the speed and quality of wound healing [117]. For instance, Yu *et al*. [123] found that insulin curbs the expression of p38, NF-κB, and STAT1 proteins when blood sugar levels are high. It does this by setting off the PI3K/Akt/Rac-1 signaling chain. As a result, it dials down the inflammatory process and supports the repair of chronic wounds. Similarly, a therapy using extracellular vesicles derived from endothelial cells enhances fibroblast proliferation and reduces cellular senescence via enhanced nuclear YAP translocation and engagement of the PI3K/Akt/mTOR pathway, accelerating skin wound healing [124].

Moreover, conventional Chinese medicine offers distinct benefits for modulating tissue repair. It can modulate multiple signaling pathways, including the Wnt, Nrf2/ARE, MAPK, PI3K/AKT, NF-κB, Notch, TGF-β/Smad, and HIF-1α/VEGF pathways, to promote the healing of diabetic wounds by regulating inflammatory balance, reducing oxidative stress, and normalizing glucose metabolism [125]. Screening for core targets of traditional Chinese medicine in the treatment of diabetic wounds and developing modern innovative drugs based on these targets are gradually becoming promising research directions.

Recent research has revealed that extracellular vesicles from MSCs pre-conditioned with pioglitazone (PGZ-Exos) significantly boost the blood vessel–forming potential of human umbilical vein endothelial cells. This effect occurs through stimulation of the PI3K/AKT/eNOS signaling cascade, ultimately accelerating the recovery of diabetic wounds. The findings highlight a promising therapeutic avenue for improving impaired wound healing in diabetic patients [126]. Similarly, Ying Zhang *et al*. [85] effectively improved lesion repair in murine diabetes models by stimulating neovascularization with drugs that activate HIF-1α. Guodong Li *et al*. [127] further developed a HIF-1α stabilizer that elevates HIF-1α levels and triggers its downstream gene activation (such as VEGF, GLUT1, and EPO), significantly accelerating wound healing in mouse models.

Various AMPK activators have positive effects on stimulating fibroblast and keratinocyte expansion and movement, enhancing angiogenesis, and exhibiting anti-inflammatory and antioxidant properties, as well as blocking RAGE signaling—functions that are highly beneficial for diabetic wound healing [96, 128]. However, further clinical research is needed to determine which AMPK activator is the most effective and whether it should be combined with other treatments for diabetic ulcers. These studies indicate that regulating glucose metabolism and signaling pathways through several strategies can significantly enhance diabetic wound recovery, laying a robust scientific groundwork for innovative treatment strategies.

#### Regulating key enzymes in glycolysis

Current therapeutic research is exploring various strategies to promote wound healing by targeting key enzymes involved in glucose metabolism, with a focus on regulating the glucose transport and glycolysis pathways to improve cellular metabolism and functionality. Haiwen Li *et al*. [129] reported that papaya extracts can stimulate GLUT2 expression, increase glucose uptake, and enhance fibroblast migration, thus effectively improving wound healing. Similarly, white copper and *Aloe vera* extracts can upregulate GLUT1 expression, promoting cell proliferation and accelerating the repair of diabetic wounds [25]. These findings indicate that regulating the expression of GLUT proteins can significantly improve the wound-healing process.

In addition to glucose transporters, targeting key enzymes in the glycolytic pathway also shows promise for promoting wound healing. Hamanaka *et al*. [130] discovered that p63 directly targets PFKFB3, enhancing glycolytic metabolism in epidermal keratinocytes. While PFKFB3 overexpression can promote cell proliferation, it also inhibits differentiation; conversely, reducing PFKFB3 expression decreases proliferation and promotes differentiation. These findings suggest that the regulation of PFKFB3 has a dual role in wound healing, warranting further research to explore its specific functions at different healing stages for optimized application.

Moreover, other studies are investigating strategies targeting rate-limiting enzymes in glycolysis, such as HK and PFK1, to promote wound healing. Activating these enzymes can increase the glycolytic rate, providing sufficient energy support for cell proliferation and tissue repair. These strategies not only help improve the metabolic state of wounds but also support wound repair by regulating the cellular energy balance.

These research suggests modulation of crucial glucose metabolism enzymes/proteins noticeably aids cutaneous regeneration. However, the regulation of these enzymes and transport proteins is complex and multifaceted, necessitating in-depth research on their specific roles in different wound environments and healing stages when these therapeutic strategies are developed. Future therapeutic research should focus on the fine-tuned regulation of these metabolic pathways and explore optimal treatment combinations and applications to provide more effective wound healing options for patients.

#### Regulating mitochondria

Enhancing mitochondrial function to promote wound healing is a key focus of current research, especially in diabetic patients. The PPARα agonist fenofibrate effectively ameliorates mitochondrial dysfunction in the corneas of diabetic mice, significantly accelerating the healing of corneal wounds [131]. This treatment strategy optimizes mitochondrial function, restores normal metabolic activity in corneal epithelial cells and aids in effective wound repair. Additionally, extracellular vesicles derived from mesenchymal stem cells (MSC-sevs) can stabilize the expression of the critical mitophagy regulator Parkin by transporting the deubiquitinating enzyme USP9, promoting mitophagy and restoring mitochondrial function, which is crucial for improving wound healing [132].

Further studies indicate that regulating mitophagy is an effective strategy for enhancing wound healing. For example, the overexpression of ANT2 can activate glycolysis and induce mitophagy, increasing ATP production and accelerating healing in aged skin wounds [133]. This mechanism offers new therapeutic avenues for addressing age-related wound healing impairments. Moreover, research has shown that directly treating wounds with mitochondria isolated from healthy cells can significantly accelerate healing by providing functional mitochondria [134]. This approach increases the energy supply and cellular function in damaged tissues, representing a novel direction in wound therapy. Mitochondrial reactive oxygen species have been identified as significant contributors to delayed wound healing. The application of mitochondria-targeted antioxidants can significantly reduce oxidative stress, thereby accelerating the closure of diabetic wounds while promoting epithelial cell and granulation tissue formation, as well as angiogenesis [135]. Enhancing mitochondrial integrity in macrophages—by reducing membrane permeability and maintaining stable membrane potential—shifts these immune cells toward an anti-inflammatory state. This transformation significantly speeds up the healing process for both skin and mucosal injuries [106]. Collectively, these studies suggest that regulating mitochondrial function through various strategies can improve the energy metabolic state of wounds and effectively modulate cellular immune responses and regenerative capabilities, providing new therapeutic insights and potential clinical applications for tissue repair in diabetic patients and those suffering from various intricate health issues.

### Prospects and challenges

Glucose catabolism is critical for cutaneous repair, fueling regenerative cells with vital energy and metabolites. It not only supports cell proliferation, migration, and functional maintenance but also influences the healing process by regulating extracellular matrix remodeling and immune responses. Current research has preliminarily indicated that modulating glucose metabolism–related pathways or targeting key metabolic enzymes, such as GLUTs, glycolytic enzymes (e.g. PFKFB3 and HK), and metabolic regulators (e.g. AMPK), can promote wound healing to some extent. However, because the healing process is highly complex, the glucose metabolic needs and characteristics of different cell types vary at different healing stages, leading to significant differences and uncertainties in targeted therapeutic outcomes.

In acute wounds, cells typically rely on glycolysis for a rapid energy supply to support proliferation and migration. In contrast, chronic or diabetic wounds often exhibit metabolic disturbances that are potentially characterized by excessive glycolysis coupled with suppressed oxidative phosphorylation or mitochondrial dysfunction, resulting in insufficient energy metabolism and exacerbated inflammatory responses. This metabolically disrupted environment can hinder normal wound healing. Therefore, precisely regulating glucose metabolic pathways to improve the homeostasis of the wound microenvironment has become an important research focus. However, achieving precise regulation of glucose metabolism across different healing stages and cell types to achieve the ideal wound microenvironment balance remains a major challenge.

Moreover, the metabolic demands for wound healing are influenced not only by the intrinsic state of the cells but also by external environmental factors such as the oxygen supply, nutritional status, and inflammatory response. The interplay among these factors makes the regulation of glucose metabolism even more complex. Thus, to really nail down more effective ways to treat wounds, we need a solid grasp of how glucose metabolism shifts and changes throughout the healing process, and how all the different metabolic pathways talk to each other.

Subsequent studies ought to concentrate on a multifaceted examination of the underlying dynamics by which glucose metabolism influences wound healing utilizing a combination of metabolomics, cellular metabolic analyses, and bioinformatics to construct a comprehensive map of the regulatory network of glucose metabolism. Additionally, individual differences and the diversity of wound types should be considered when developing personalized and targeted interventions. Combining the application of cutting-edge technologies such as CRISPR gene editing and smart responsive materials in wound therapy, targeted metabolic gene regulation and dynamic drug delivery systems will be further be improved. Through these efforts, new theoretical foundations and drug development directions are anticipated to emerge, advancing the treatment of wound healing.

## Conclusions

Glucose metabolism significantly affects tissue repair, impacting hemostasis, inflammation, cell growth, and matrix restructuring. Normal glucose metabolism supplies cells with essential energy and metabolic intermediates that support cell proliferation, migration, and functional maintenance. However, in chronic wounds—particularly those associated with diabetes—abnormal glucose metabolism can lead to increased oxidative stress, excessive inflammatory responses, and mitochondrial dysfunction, which collectively delay wound healing. A deeper understanding of the specific mechanisms of glucose metabolism, including the roles of key enzymes and signaling pathways, can help reveal the pathological basis of wound healing disorders. Intervention strategies targeting glucose metabolism, such as regulating the activity of key metabolic enzymes and improving mitochondrial function, offer promising avenues for new treatments. Future studies must delve into the intricacies of glucose metabolism across various cell varieties and healing phases, in a bid to concoct more potent metaboregulatory approaches. Such an endeavor aims to accelerate the healing process and enhance the overall well-being of patients.
